# Photon-counting detector CT virtual monoenergetic imaging for bone mineral density quantification: Validation with Micro-CT

**DOI:** 10.1016/j.isci.2026.115197

**Published:** 2026-03-03

**Authors:** Yuanbo Ma, Yaman Li, Danyang Su, Yufang Du, Ke Qi, Simeng Wang, Hao Shen, Mengze Ma, Fei Li, Shenyu Yang, Qiuju Miao, Xiaopeng Yang

**Affiliations:** 1 The First Affiliated Hospital of Zhengzhou University, Zhengzhou 450052, China

**Keywords:** Medical imaging

## Abstract

Volumetric bone mineral density (vBMD) is crucial for evaluating bone quality. We established rabbit tibial defect models to assess virtual monoenergetic imaging (VMI) based on photon-counting detector CT (PCD-CT) for vBMD quantification, using micro-CT as the reference standard. VMI-derived CT values strongly correlated with micro-CT–measured vBMD across all energies (*r* > 0.925). The 50 keV VMI achieved the highest correlation with micro-CT (*r* = 0.951) and the best model fit (*R*^2^ = 0.9138), with low prediction errors (MAE = 0.0114 g/cm^3^; RMSE = 0.0135 g/cm^3^). In terms of image quality, the signal-to-noise ratio (SNR) and contrast-to-noise ratio (CNR) were superior at 70 keV. Overall, the energy range of 60–80 keV provided the optimal balance between predictive accuracy and image quality. PCD-CT is poised to play a transformative role in orthopedic research and clinical bone health management by enabling accurate, standardized, and longitudinal quantitative assessment.

## Highlights

• Accurate *in vivo* vBMD quantification with PCD-CT VMI
• Phantom-free and highly reproducible method for standardized vBMD assessment
• Enables *in vivo* monitoring in large animals for translational research

## Graphical abstract

**Figure:**
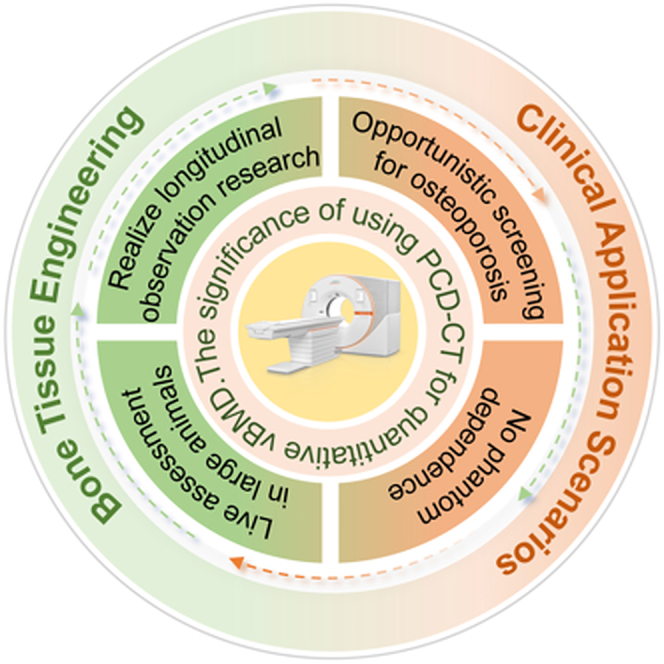

## Introduction

Quantitative assessment of bone mineral density (BMD) plays a critical role, particularly in osteoporosis,^1^ fracture risk prediction, post-joint replacement assessment, and analysis of the osseointegration performance of implant materials. The World Health Organization has identified the reduction of BMD as one of the diagnostic criteria for osteoporosis, and it is significantly associated with the risk of fractures.^2^ While dual-energy X-ray absorptiometry (DXA) is the most common screening tool,^3^ it is limited by poor spatial resolution and its two-dimensional nature, which prevent detailed analysis of bone microstructure.^4^ In contrast, quantitative CT (QCT) and micro-CT can provide better three-dimensional structural information and are widely used in bone density analysis.^5^ Micro-CT, in particular, has become the gold standard in preclinical animal model research.^6^ However, QCT increases the complexity of bone density quantification as it requires calibration of the phantom, making it difficult to carry out routinely.^7^ Although micro-CT has an extremely high resolution, it is limited by the size of its examination chamber and can only be applied to small animals and *in vitro* specimens, lacking the ability for clinical transformation.^8^^,^^9^^,^^10^ Therefore, developing a standardized, quantitative, and clinically translatable imaging method for BMD assessment remains a critical goal.

The recent clinical approval and adoption of photon-counting detector CT (PCD-CT) represents a significant advance in medical imaging.^11^ Unlike conventional energy-integrating detector CT (EID-CT), PCD-CT is equipped with a photon-counting detector based on semiconductor material, which can directly convert incident X-rays into electrical signals through charge separation. The indirect detection process relying on scintillator and photomultiplier in traditional system is skipped.^12^ This enables PCD-CT to count each incident X-ray photon separately and classify it according to its energy, thereby achieving the normalization of energy spectrum imaging in a true sense.^13^ Unlike dual-energy CT which requires a preset scanning mode, the raw data obtained by PCD-CT naturally contains energy information, allowing for the reconstruction of virtual monoenergetic images (VMIs) at any energy level from a single scan.^14^ In addition, by eliminating traditional reflection devices, PCD offers higher geometric efficiency, allowing for smaller detector sizes and greatly improving spatial resolution.^15^ This advantage is particularly critical for bone structure assessment, providing a new possibility for the quantitative analysis of vBMD and the integration of bone implants.^11^ More importantly, the virtual monoenergetic images output by PCD-CT have highly consistent energy spectrum characteristics. At a fixed energy level (such as 70 keV), its CT value maintains excellent stability and comparability under different scanning parameters (such as tube voltage kVp, tube current mAs),^16^ size differences, or whether it is enhanced or not. This spectral standardization feature significantly improves the reproducibility of CT values and provides a solid technical foundation for quantitative bone mineral density assessment across platforms, individuals, and time points.^17^

Although previous studies have shown that photon-counting CT-based VMI has good image quality and quantitative stability,^17^ there was a lack of studies systematically evaluating the performance of VMI in volumetric bone mineral density (vBMD) quantification in animal models. Particularly in scenarios involving biomaterial implantation, bone regeneration, and osseointegration, the sensitivity and reliability of VMI at different energy levels for quantification of vBMD still need to be further verified.

Therefore, this study is designed to systematically evaluate the sensitivity and accuracy of VMI of PCD-CT at different energy levels (40–110 keV) for quantitative vBMD, and to compare and verify the vBMD values obtained by high-resolution micro-CT as the gold standard. To this end, we constructed a New Zealand rabbit tibial plateau defect model and implanted 3D printed polyetheretherketone (PEEK) scaffolds with different osseointegration properties to simulate the common clinical bone repair situation. The CT value, signal-to-noise ratio (SNR), and contrast-to-noise ratio (CNR) at each energy level were measured, and the correlation and consistency between them and vBMD measured by micro-CT were analyzed. This study aims to evaluate the feasibility of VMI reconstruction based on PCD-CT for vBMD quantification using an animal model system. Its performance was verified by comparison with the gold standard micro-CT to screen out the most suitable reconstruction energy level for quantitative evaluation, and to provide a theoretical basis for its application in quantitative vBMD.

## Results

### Comparison of image parameters at different energy levels

**Figure 1:**
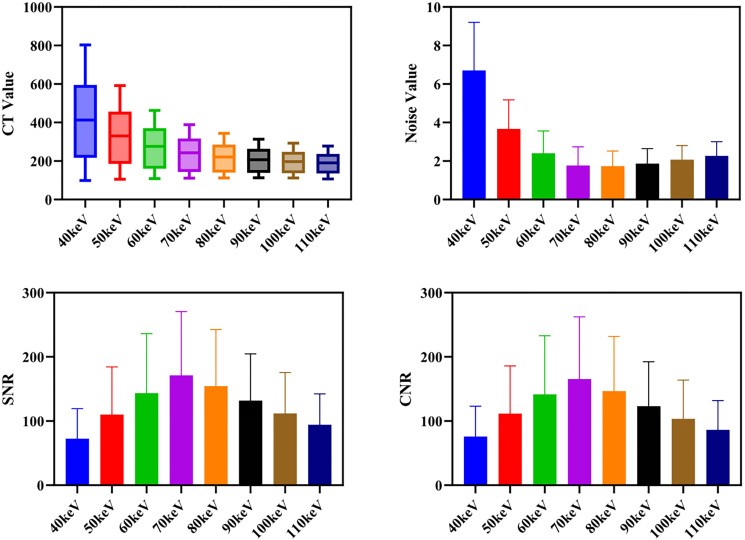
Comparison of image parameters (HU, Noise, SNR, CNR) at different virtual monoenergetic energy levels Box-and-whisker plots show the median (center line), interquartile range (box), and minimum to maximum values (whiskers). For bar plots, data are represented as mean ± SD.

As shown in Figure 1, with the increase in virtual monoenergetic levels, the average CT value (HU) of the target area shows a gradual downward trend, and the range of CT values gradually narrows. Statistical results show that CT values did not vary significantly between a given energy level and the two adjacent energy levels immediately above and below it (*p* > 0.05), but there are significant differences between non-adjacent energy levels (*p* < 0.001).

In the 60–110 keV range, the overall image noise level is low (Figure 1), and there are no significant differences between energy levels (*p* > 0.05). However, in the low-energy range of 40–50 keV, the noise value increased significantly. The lower the energy level, the more obvious the increase in noise, showing statistical significance (*p* < 0.05).

SNR remained relatively high in the 60–90 keV range (Figure 1), with no significant differences between groups (*p* > 0.05). As the energy level continues to rise or fall, the SNR value decreases significantly. A comprehensive comparison revealed that the SNR value was highest at the 70 keV energy level, demonstrating the best performance.

The trend of CNR is consistent with that of SNR (Figure 1), performing well between 60 and 90 keV, with no significant differences between groups (*p* > 0.05); however, at higher or lower energy levels, CNR decreases significantly, with statistically significant differences (*p* < 0.05). Similarly, 70 keV is the optimal energy level for CNR performance.

The detailed *p*-values are summarized in Table 1.

**Table 1:** Adjusted *p*-values of pairwise comparisons between different keV levels for HU, noise, SNR, and CNR

Comparison(keV)	*p*-value (HU)	*p*-value (Noise)	*p*-value (SNR)	*p*-value (CNR)
40 keV vs. 50 keV	0.999	0.752	0.033	0.0172
40 keV vs. 60 keV	0.081	0.000	0.000	0.000
40 keV vs. 70 keV	0.000	0.000	0.000	0.000
40 keV vs. 80 keV	0.000	0.000	0.000	0.000
40 keV vs. 90 keV	0.000	0.000	0.000	0.000
40 keV vs. 100 keV	0.000	0.000	0.009	0.063
40 keV vs. 110 keV	0.000	0.000	0.999	0.999
50 keV vs. 60 keV	0.999	0.021	0.053	0.105
50 keV vs. 70 keV	0.096	0.000	0.000	0.000
50 keV vs. 80 keV	0.000	0.000	0.000	0.006
50 keV vs. 90 keV	0.000	0.000	0.296	0.999
50 keV vs. 100 keV	0.000	0.000	0.999	0.999
50 keV vs. 110 keV	0.000	0.010	0.999	0.235
60 keV vs. 70 keV	0.999	0.320	0.980	0.999
60 keV vs. 80 keV	0.068	0.186	0.999	0.999
60 keV vs. 90 keV	0.000	0.999	0.999	0.999
60 keV vs. 100 keV	0.000	0.999	0.172	0.030
60 keV vs. 110 keV	0.000	0.999	0.000	0.000
70 keV vs. 80 keV	0.999	0.999	0.999	0.999
70 keV vs. 90 keV	0.081	0.999	0.218	0.201
70 keV vs. 100 keV	0.000	0.999	0.000	0.000
70 keV vs. 110 keV	0.000	0.571	0.000	0.000
80 keV vs. 90 keV	0.999	0.999	0.999	0.999
80 keV vs. 100 keV	0.124	0.999	0.002	0.001
80 keV vs. 110 keV	0.001	0.344	0.000	0.000
90 keV vs. 100 keV	0.999	0.999	0.804	0.571
90 keV vs. 110 keV	0.124	0.999	0.000	0.000
100 keV vs. 110 keV	0.999	0.999	0.860	0.656

### Correlation between CT values and vBMD at different energy levels

**Figure 2:**
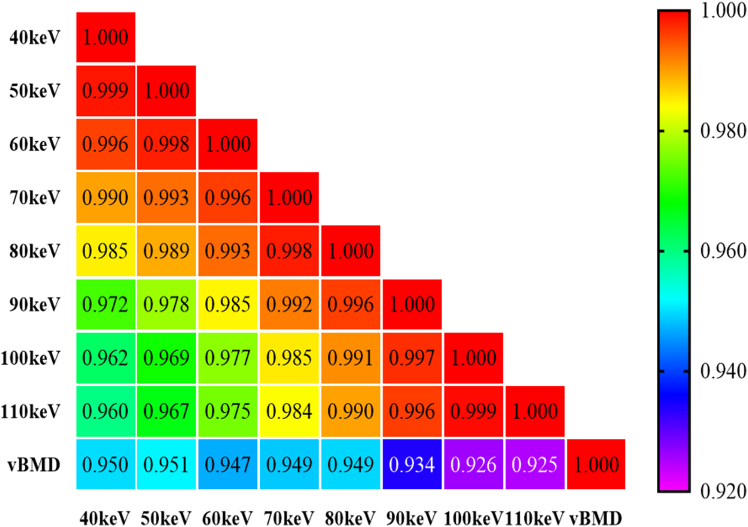
Spearman’s correlation heatmap shows the correlation between CT values and vBMD values in the target area at different VMI levels The closer the color is to red, the higher the correlation.

The Spearman rank correlation analysis method was used to assess the correlation between CT values and measured vBMD at different virtual monoenergetic energy levels (40–110 keV), and the results are shown in Figure 2. The CT values of the target region at all energy levels showed a significant positive correlation with vBMD, with correlation coefficients (*r*) ranging from 0.925 to 0.951. Higher correlations were observed in the 40–80 keV range, with the highest correlation between CT values and vBMD at 50 keV (*r* = 0.951). As the energy level increases, the correlation decreases slightly but remains at a high level (*r* > 0.92). This result indicates that CT values in VMI have good potential for quantitative vBMD measurement. Among these, the medium-to-low energy range (40–80 keV) may be the optimal energy level selection range.

### Linear regression analysis

**Figure 3:**
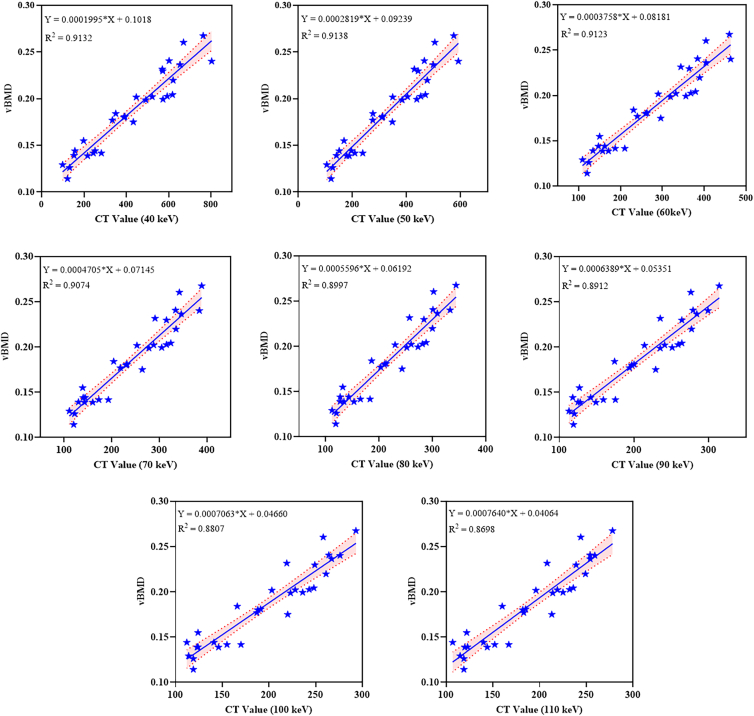
Linear regression results of CT values and vBMD at different energy levels The X axis represents CT values (HU) measured at different energy levels, and the Y axis represents vBMD (g/cm^3^) measured by micro-CT. Each scatter point represents a paired measurement of PCD-CT–derived CT value and micro-CT–derived vBMD for a single defect site. The regression equation and *R*^2^ value are labeled in each figure to evaluate the model’s fitting ability.

A simple linear regression analysis was performed on CT values and vBMD values at different energy levels, and the results are presented in a scatterplot (Figure 3). The regression equation and coefficient of determination (*R*^2^) are shown in each graph to evaluate the explanatory power of different energy level CT values for vBMD.

The results showed that CT values at different energy levels exhibited a good linear relationship with vBMD (*R*^2^: 0.8698–0.9138). Among these, 50 keV exhibited the highest coefficient of determination (*R*^2^ = 0.9138), with the linear regression equation being Y = 0.0002819∗X + 0.09239. In contrast, as the energy level increased, the coefficient of determination decreased slightly, which may be related to a decrease in tissue contrast.

### Performance evaluation of prediction models

To assess the predictive performance of CT values at different VMI levels for vBMD, this study conducted regression analysis and multi-indicator assessment of predicted values and actual values. The predicted values were the predicted vBMD generated by 5-fold cross-validation at different levels, and the actual values were the vBMD measured by micro-CT.

**Figure 4:**
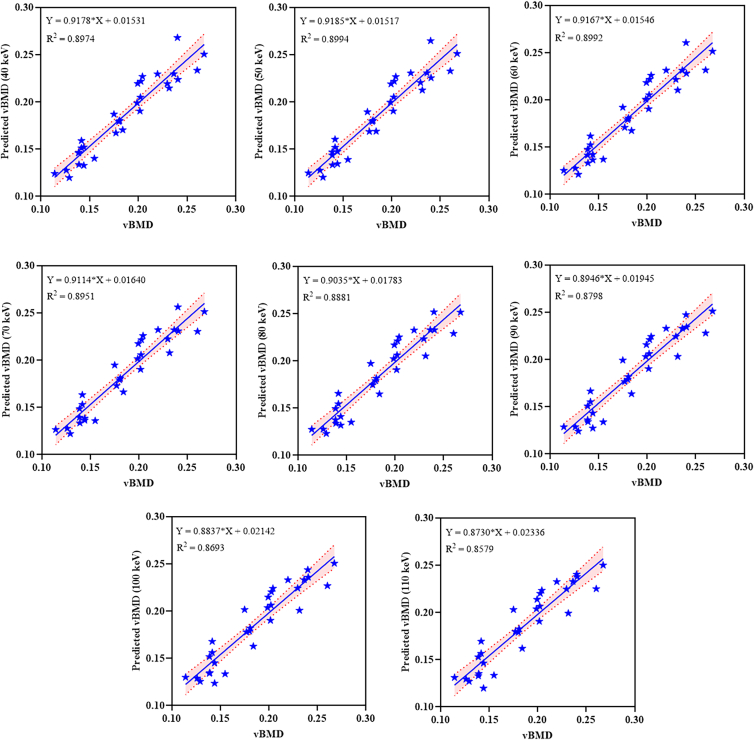
Linear regression analysis of predicted bone mineral density values and measured values at different energy levels The figure shows the linear relationship between the predicted vBMD values based on 5-fold cross-validation and the measured vBMD values obtained by micro-CT. The X axis represents the vBMD measured by micro-CT, and the Y axis represents the predicted vBMD values at different energy levels. Each scatter point represents a paired measurement of predicted and measured vBMD from a single defect site. The regression equation and coefficient of determination (*R*^2^) are labeled in the figure to assess the goodness of fit of the prediction model.

As shown in Table 2, the MAE varies little across energy levels, ranging from 0.01125 to 0.01247. Among these, the MAE performs best at 60 keV. Both MSE and RMSE show minimum values at 50 keV (0.00018 and 0.01353, respectively), suggesting that this energy level has better prediction accuracy. A regression analysis was further conducted between the predicted vBMD and the actual vBMD, with the results shown in Figure 4. The predicted values at all energy levels exhibited a good linear relationship with the actual values, with *R*^2^ values ranging from 0.8579 to 0.8994, with the highest *R*^2^ value observed at 50 keV (*R*^2^ = 0.8994). The regression slope is close to 1, indicating that the predicted results are closely aligned with the actual values, demonstrating high fitting accuracy.

**Table 2:** Performance evaluation indicators of vBMD prediction models at different energy levels

Energy Levels(keV)	MAE	MSE	RMSE	*R*^2^
40	0.011609363	0.0001868	0.013668732	0.8974
50	0.011390837	0.0001831	0.013531972	0.8994
60	0.011254253	0.0001835	0.013545992	0.8992
70	0.0113745	0.0001908	0.013811827	0.8951
80	0.011576913	0.0002035	0.014264164	0.8881
90	0.011780373	0.0002184	0.014779786	0.8798
100	0.01212621	0.0002375	0.015411247	0.8693
110	0.01247122	0.0002583	0.01607044	0.8579

Overall, 50 keV performed the best in all evaluation metrics, with the highest goodness-of-fit (*R*^2^ = 0.8994) and lowest error values (MSE = 0.00018, RMSE = 0.01353), making it the optimal energy level for predicting vBMD performance in this study. Overall, the differences in various indicators within the low-to-medium energy range (40–70 keV) are relatively small, and the predictive performance is relatively stable. However, at higher energy levels, the predictive performance gradually declines, suggesting that it may be more advantageous to prioritize low-to-medium energy levels when performing the quantitative analysis of vBMD based on VMI.

### Consistency analysis

**Figure 5:**
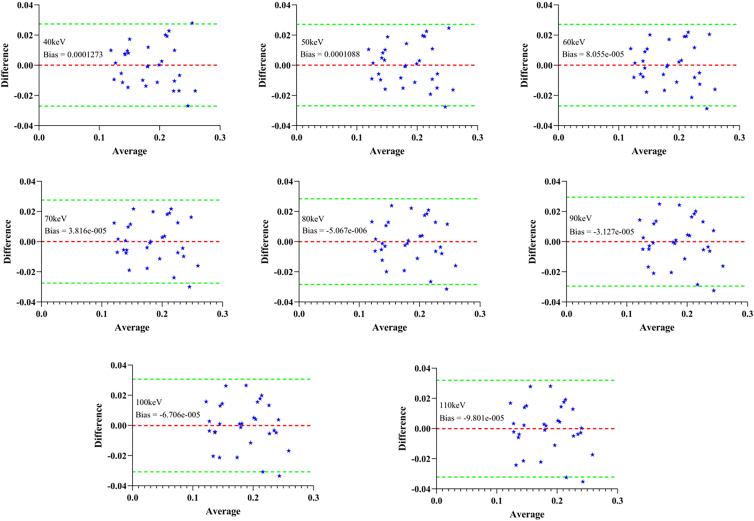
Bland–Altman plot of predicted vBMD versus micro-CT measured vBMD at different energy levels The red dotted line represents the mean bias, and the green dotted lines above and below represent the 95% limits of agreement of ±1.96 standard deviations.

The Bland–Altman plot was further used to assess the consistency between the predicted values and the true values, and the average bias and 95% limits of agreement at each energy level are shown (Figure 5). The results show that the differences between the predicted values and the actual values at all energy levels are randomly distributed, with no obvious systematic bias. The bias values at each energy level are close to 0 (Bias: −0.00009801–0.0001273 g/cm^3^), indicating that the predicted results are highly consistent with the actual results.

### Additional validation in vertebral specimens

#### Correlation between CT values and vBMD

Pearson’s correlation analysis was performed to evaluate the relationships among CT values at different virtual monoenergetic energy levels (40–80 keV) and the vBMD measured by micro-CT, as illustrated in Figure S1. Overall, all CT values across the evaluated energy levels showed strong positive correlations with vBMD (r = 0.97–0.99). The correlation at 40 keV was the highest (r = 0.99), and it decreased slightly as the energy level increased. However, it still remained at a relatively high level throughout.

#### Linear regression

A simple linear regression analysis was performed on CT values and vBMD values at different energy levels, and the results are presented in a scatterplot (Figure S2). The results showed that CT values and vBMD values at different energy levels were significantly linearly correlated.

#### Consistency analysis

The Bland–Altman plot was further used to evaluate the agreement between the predicted vBMD values and the reference values obtained from micro-CT (Figure S3). The mean bias and the 95% limits of agreement at each energy level are displayed. The results showed that a small mean bias between the predicted and micro-CT–measured vBMD values, with all data points falling within the 95% limits of agreement, indicating acceptable agreement between the two measurements. Notably, the regression model established at 50 keV exhibited the smallest mean bias (bias = −0.0004497 g/cm^3^), whereas a gradual increase in deviation was observed at higher energy levels.

## Discussion

This study confirmed the feasibility and high accuracy of quantitative evaluation of vBMD based on VMI of PCD-CT using a rabbit tibial defect model. It was found that the CT values of the region of interest in PCD-CT images reconstructed at different energy levels were highly and positively correlated with the vBMD measured by high-resolution micro-CT. In the range of 40–110 keV, the CT values of each single-energy plane reflected the actual vBMD well, and the correlation coefficient *r* remained above 0.925. Among them, the low and medium energy levels (40–80 keV) performed the best. The highest correlation between CT values and vBMD was found in the 50 keV VMI (*r* = 0.951). With the increase of energy level, the correlation decreased slightly, but still maintained at a high level (*r* > 0.925). This suggests that the VMI energy selection provided by PCD-CT is critical for vBMD quantification and that the quantitative accuracy of PCD-CT is comparable to micro-CT at appropriate energy levels, such as 40 to 80 keV. In fact, a recent study^18^ comparing PCD-CT and HR-pQCT (high resolution peripheral quantitative CT) for vBMD measurement in phantoms and *ex vivo* samples showed a correlation of more than 0.98. Our results are consistent with reports and support the use of PCD-CT as an alternative for *in vivo* vBMD assessment.

In this study, we comprehensively assessed the predictive performance and stability of CT values for vBMD. The results of Spearman’s analysis showed a significant positive correlation between CT values and vBMD at all energy levels, validating the monotonic relationship between CT values and vBMD. On this basis, the simple linear regression models constructed at different energy levels all showed high fit. The coefficient of determination *R*^2^ ranged from 0.8698 to 0.9138, indicating that the CT value in VMI could explain most of the vBMD variation. The regression model at 50 keV had the best fitting effect (*R*^2^ = 0.9138), suggesting that the CT value at this energy level was the most accurate in predicting vBMD. In addition, a 5-fold cross validation method was introduced to evaluate the prediction stability and generalization ability of the model under different energy levels. The results show that the predicted vBMDs from the 5-fold cross-validation are in good agreement with the true values measured by micro-CT, with the average deviation close to 0 (Bias: −0.00009801–0.0001273 g/cm^3^) and the majority of the points fall within the 95% agreement limits. The overall *R*^2^ value ranged from 0.8579 to 0.8994, and 50 keV had the best performance (*R*^2^ = 0.8994), further confirming the stable linear relationship between CT value and vBMD in VMIs. Despite the limited sample size, the assessment of the stability of the prediction model by cross-validation enhanced our confidence in the reliability of the results.

Interestingly, while 50 keV provided the highest vBMD correlation and prediction accuracy, image quality metrics (SNR and CNR) performed best at a slightly higher energy range (70 keV). This suggests that lower energy improves the sensitivity to detect differences in vBMD, but at the same time increases image noise. However, the moderate energy range (60–80 keV) achieves a perfect balance at high SNR. In fact, we found that the 60–80 keV energy segment performed well in both accuracy and image quality metrics, indicating that this energy range is most suitable for vBMD quantification analysis.

In the clinical context, vBMD quantification by PCD-CT has many advantages over traditional techniques. Due to the limitation of imaging principle, traditional DXA can only provide two-dimensional surface density and is easily affected by overlapping tissues, which cannot reflect the changes of trabecular structure and volume density.^19^ QCT is more accurate than DXA for osteoporosis diagnosis and fracture risk assessment because it provides three-dimensional vBMD.^20^ However, QCT usually requires the placement of a calibration phantom during scanning, increasing operational complexity and sources of error. In addition, different CT devices and scanning parameters can lead to a lack of comparability of QCT measurements, requiring cross-device calibration to ensure consistency.^3^^,^^21^ In contrast, because the detector principle of PCD-CT is different from that of traditional energy integration detectors, “energy spectrum imaging normalization” can be achieved, that is, multienergy information can be obtained in a single scan without prior setup.^22^ This allows the researcher to reconstruct monoenergetic images at arbitrary energy levels post hoc, allowing us to compare the scan results of different individuals and different times at a fixed energy level without the influence of the scan parameters.^23^^,^^24^^,^^25^ Previous studies have shown that the VMI of PCD-CT can significantly reduce the influence of different patient body types on CT values and improve quantitative accuracy and consistency.^26^ This regularization of energy spectrum and excellent imaging stability mean that PCD-CT can achieve comparable results without relying heavily on phantom calibration as traditional QCT.^26^ Therefore, PCD-CT is expected to enable longitudinal quantitative studies across different time points, allowing direct comparison of results over time and facilitating deeper and long-term investigations of bone regeneration.

Notably, the advantages of PCD-CT open new avenues for opportunistic screening for osteoporosis, enabling routine clinical scans to be simultaneously used for diagnostic imaging and bone quality assessment. For example, during pulmonary nodule screening, vertebral vBMD could be opportunistically assessed using the simultaneously acquired spectral information. This approach is expected to improve osteoporosis detection rates,^27^ which is particularly important given the aging population. A recent clinical study has demonstrated that scout images based on PCD-CT can quantify aBMD of the lumbar spine region, and the results are highly consistent with those of DXA (bias = −0.57%).^28^ These findings highlight the promise of PCD-CT in opportunistic screening for osteoporosis. By reconstructing and analyzing existing data, PCD-CT can detect bone changes in individuals not undergoing dedicated BMD screening. This helps overcome the compliance challenges associated with traditional DXA,^27^ which requires patients to actively present for screening. In the future, the addition of BMD assessment to routine CT examination is expected to greatly improve the coverage of osteoporosis screening and achieve early intervention for high-risk populations.

In addition to its potential application in osteoporosis screening, PCD-CT has also shown good prospects in bone tissue engineering research. Instead of using a homogeneous calibration phantom under ideal conditions, 3D printed PEEK scaffolds with different osteoinductive abilities were introduced as implants in this study, which showed different degrees of bone growth in the animal model *in vivo*, thus simulating the complex and dynamic bone regeneration environment in clinical practice. This design surpasses the limitations of traditional phantom research and makes this study closer to practical application scenarios. The results showed that PCD-CT could accurately reflect the difference of new bone mass in different PEEK scaffolds, and the vBMD values measured by PCD-CT were highly consistent with those measured by micro-CT, which reflected the growth quality of bone tissue on the implant surface to a certain extent. These results suggest that PCD-CT is not only suitable for osteoporosis screening but also can be used as an effective tool to evaluate the osseointegration performance of implants *in vivo*, which provides a non-invasive and efficient imaging method for the development and verification of orthopedic implant materials.

To further verify the applicability of PCD-CT for quantitative vBMD assessment in clinically relevant skeletal regions, an additional validation was performed on rabbit vertebral bodies (L1–L4) at 40–80 keV. Correlation analysis showed a strong relationship between CT values obtained by PCD-CT and vBMD measured by micro-CT (*r* = 0.97–0.99), consistent with the findings from the tibial defect model (Figure S1). The scatterplots demonstrated strong linear relationships between PCD-CT–derived CT values and micro-CT–measured vBMD (Figure S2). The CT values from the vertebral samples were input into the regression equations established in this study to obtain the predicted vBMD values. Subsequently, Bland–Altman analysis was performed to assess the agreement between the predicted and reference vBMD values. The mean absolute bias ranged from 0.0004497 to 0.0194 g/cm^3^, indicating minor differences between the predicted and measured vBMD values (Figure S3). However, except for 40 keV, all other energy levels exhibited a slight tendency toward underestimation. This underestimation may be attributed to the relatively narrow vBMD range of the training data, which mainly consisted of low-density samples from the tibial defect model, leading to systematic underestimation in higher-density regions. This energy-dependent underestimation may also be related to differences in marrow composition between the tibia and the spine. These findings suggest that future large-scale or clinical prediction models should include a wider range of bone densities and multiple skeletal sites to improve model accuracy and generalizability. In addition, the regression model constructed at 50 keV showed the smallest bias between predicted and measured values, while the bias gradually increased with higher energy levels. This observation is consistent with the finding that the 50 keV energy level achieved the highest correlation, best goodness of fit, and lowest RMSE in this study. Taken together, these results support the potential applicability of PCD-CT for quantitative vBMD assessment beyond the specific tibial defect model.

For the selection of an image reconstruction algorithm, the reconstruction kernel (Qr76) with high image quality and spatial resolution was used in this study to identify and measure the target area more accurately. Compared with the reconstruction kernel with low spatial resolution, the high-resolution algorithm can improve the accuracy and repeatability of ROI selection, so as to better meet the requirements of ROI location consistency in the comparison of results between different methods.^29^ In clinical applications, high resolution algorithms are recommended if there is a need to focus on microstructural changes and to ensure a precise range of ROI selection.^30^ In quantitative analysis scenarios that do not require high resolution, choosing a low spatial resolution reconstruction kernel may improve quantitative accuracy.

Overall, this study validates the feasibility and accuracy of PCD-CT VMI-based vBMD quantification in an animal bone defect model. The high degree of consistency with micro-CT, the gold standard, particularly at low-to-mid energy levels, establishes PCD-CT as a reliable and standardized assessment tool. Future studies should focus on extending this method to clinical populations and exploring the value of PCD-CT BMD assessment in multi-center studies and longitudinal follow-up. Owing to its high resolution, spectral regularization, and lack of calibration, PCD-CT is poised to play a transformative role in orthopedic research and clinical bone health management.

### Limitations of the study

There are still some limitations of this study. The evaluation object of this study was an animal model, which is different from humans, so the quantitative results in this study need to be applied with caution when directly applying to the clinic. While we validated the accuracy of PCD-CT measurements by comparison with micro-CT, further studies involving large patient cohorts are needed to confirm the reliability and clinical applicability of PCD-CT for vBMD assessment. Moreover, previous studies have shown that PCD-CT can provide stable CT values through VMI, but these validations were all performed on a single scanner. To enable multicenter quantitative studies, further verification of the consistency and reproducibility of results across different devices is still required. Although this study used methods such as 5-fold cross validation to reduce the uncertainty in the statistics of small sample sizes, a larger sample size would enhance the robustness of the conclusions.

## Resource availability

### Lead contact

Further information and requests for resources and reagents should be directed to and will be fulfilled by the Lead Contact, Xiaopeng Yang (13837141925@163.com).

### Materials availability

This study did not generate new unique reagents.

### Data and code availability

• All data and code reported in this article will be shared by the lead contact upon request.
• This article does not report the original code.
• Any additional information required to reanalyze the data reported in this article is available from the lead contact upon request.

## Acknowledgments

We thank all participants for taking part in this study. This work was supported by the 10.13039/501100017700Henan Province Science and Technology Research Project [grant numbers 242102230117, 25210223011]; Henan Province's Key R&D Special Projects [grant number 251111311000]; Henan Province Young and Middle-aged Health Science and Technology Innovation Talent Project [grant number JQRC2024004]; and the Joint Construction Project of Henan Provincial Medical Science and Technology Research Program [grant number LHGJ20220396].

## Author contributions

Conceptualization, Y.M., S.Y., Q.M., and X.Y.; methodology, Y.M., Y.L., D.S., and Y.D.; software, K.Q. and S.W.; validation, H.S. and M.M.; formal analysis, Y.M. and D.S.; investigation, Y.M., Y.L., D.S., Y.D., and K.Q.; data curation, Y.M. and S.W.; visualization, Y.M. and M.M.; resources, F.L. and S.Y.; supervision, S.Y., Q.M., and X.Y.; project administration, S.Y. and X.Y.; funding acquisition, X.Y.; writing – original draft, Y.M.; writing – review and editing, S.Y., Q.M., and X.Y.

## Declaration of interests

The authors declare no competing interests.

## STAR★Methods

### Key resources table

**Table:** 

REAGENT or RESOURCE	SOURCE	IDENTIFIER
**Chemicals, peptides, and recombinant proteins**		

Sodium pentobarbital	Sigma-Aldrich	Cat#P3761
Xylazine hydrochloride	Selleckchem	Cat#S2516
Povidone-iodine	Sigma-Aldrich	Cat#Y0000466
Paraformaldehyde	Sigma-Aldrich	Cat#441244
Poly-L-lysine	Macklin Biochemical Co., Ltd.	Cat# P885950
ZnSO_4_·7H_2_O	Macklin Biochemical Co., Ltd.	Cat# Z820817

**Experimental models: Organisms/strains**		

New Zealand White rabbit	Weifang Saintnuo Laboratory Animal Breeding Co., Ltd	N/A RIID(NCBI_TaxID_9986)

**Software and algorithms**		

DataViewer, version 1.7.0.1	Bruker	https://www.bruker.com/en/products-and-solutions/preclinical-imaging/micro-ct/3d-suite-software.html
CTAn, version 1.23.0.2+	Bruker	https://www.bruker.com/en/products-and-solutions/preclinical-imaging/micro-ct/3d-suite-software.html
GraphPad Prism, version 10.1.2	GraphPad Software, Inc.	https://www.graphpad.com/features

**Other**		

SkyScan 1276 micro-CT scanner	Bruker	N/A
PCD-CT scanner	Siemens Healthineers	N/A
syngo.via	Siemens Healthineers	N/A (installed software)
Imaging data	This study	Available from the lead contact upon request
Code	This study	This paper does not report original code
Additional information for data reanalysis	This study	Available from the lead contact upon request

### Experimental model and study participant details

#### Animal experiment

All animal experiments were performed in compliance with institutional guidelines and international standards for the care and use of laboratory animals. Ethical approval for all experimental procedures was obtained from the Experimental Animal Ethics Committee of Henan University of Chinese Medicine (Approval No. DWLL2022040162, April 30, 2022). Every effort was made to minimize animal suffering and to ensure humane treatment in accordance with the 3Rs principles (Replacement, Reduction, and Refinement). The tibial defect model was established using healthy male New Zealand rabbits (12 weeks old, approximately 2.5 kg in weight) obtained from Weifang Saintnuo Laboratory Animal Breeding Co., Ltd. Rabbits were housed individually in stainless-steel cages under controlled conditions (15–25°C, quiet, well-ventilated, away from direct sunlight), with *ad libitum* access to certified rabbit chow and bottled drinking water. Bedding was changed daily, and the room was disinfected once per day using rotating disinfectants (hydrogen peroxide, chlorine effervescent tablets, peracetic acid, or new germicidal disinfectant) to maintain a clean environment.

Rabbits were randomly assigned to two experimental time points (4 and 8 weeks), with eight animals per time point. Each rabbit received bilateral standardized cylindrical defects (8 mm in diameter and 3 mm in depth) in the tibial plateau, resulting in 32 defects in total. The defect sites were randomly allocated to five scaffold groups, ensuring three replicates per group at each time point. Because 5 groups × 2 time points × 3 replicates = 30 samples, two redundant sites were excluded from statistical analysis. A sterilized porous PEEK scaffold (8 mm × 3 mm, 400 μm pore size), fabricated by fused deposition modeling (FDM) and subsequently surface-modified, was implanted into the defect. Medical-grade PEEK was processed into cylindrical scaffolds, sulfonated with concentrated sulfuric acid to obtain SPEEK, then coated with poly-L-lysine (PL) via a catechol-mediated reaction to form PL-SP scaffolds. PL-SP scaffolds were immersed in ZnSO_4_·7H_2_O solutions (0.5, 1.0, 1.5 mg/mL) to obtain Zn25/PL-SP, Zn50/PL-SP, and Zn75/PL-SP variants. The wound was then closed layer by layer with sutures, and the rabbits were allowed to recover.

Prior to surgery, the rabbits were anesthetized with 3% sodium pentobarbital (1 mL/kg) and xylazine hydrochloride (0.1 mL/kg). After shaving the hair on the hind limbs, the surgical area was cleaned and disinfected with povidone-iodine. The skin was incised, and a defect measuring 8 mm in diameter and 3 mm in depth was created on the tibial plateau using an 8 mm trephine drill. A sterilized 3D-printed PEEK scaffold was then implanted into the defect. The wound was then closed layer by layer with sutures, and the rabbits were allowed to recover.

At the designated time points of 4 and 8 weeks, the rabbits were euthanized with an overdose of sodium pentobarbital. The tibiae containing the implants were then carefully harvested and fixed in 4% paraformaldehyde, and scanning was performed as soon as possible after specimen collection to minimize potential tissue alterations.

#### Additional vertebral validation experiment

A total of three adult New Zealand white rabbits (12 weeks old, approximately 2.5 kg in weight) were used for this supplementary validation experiment. After euthanasia with an overdose of anesthesia, vertebral bodies from L1 to L4 were harvested (n = 12 vertebrae) for imaging analysis. All procedures were approved by the Animal Ethics Committee and conducted in accordance with institutional guidelines.

### Method details

#### Micro-CT scanning

**Figure 6:**
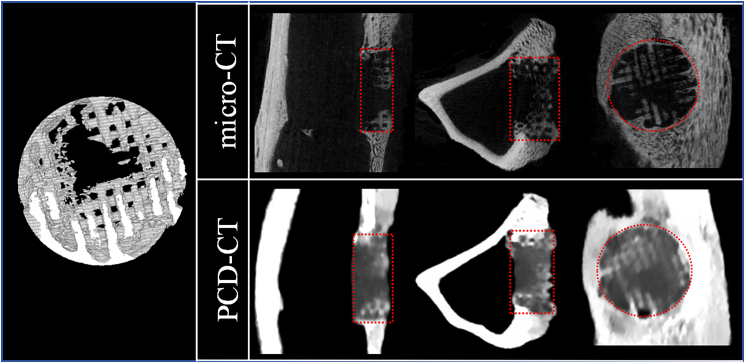
Demonstration of micro-CT and PCD-CT images illustrates the region of interest (ROI) Red dashed boxes and circles indicate the approximate extent of the bone defect in coronal, sagittal, and axial views. The left panel shows the three-dimensional reconstruction of the defect area.

Samples were scanned using micro-CT (SkyScan 1276, Bruker, Kontich, Belgium) at a pixel size of 26 μm, and the raw data were reconstructed for further analysis. The reconstructed data were imported into DataViewer (version 1.7.0.1, Bruker, Kontich, Belgium) for image adjustment to facilitate subsequent analysis. In CTAn (version 1.23.0.2+, Bruker, Kontich, Belgium), regions of interest (ROIs; 8 mm in diameter and 3 mm in thickness, aligned with the implanted scaffold position) were accurately delineated for vBMD analysis (Figure 6).

Prior to sample scanning and analysis, two hydroxyapatite (HA) phantoms with known densities (0.25 g/cm^3^ and 0.75 g/cm^3^) were scanned under the same imaging parameters as the experimental samples to establish the calibration curve. The mean grayscale values of the phantoms were used to generate a linear calibration equation between grayscale intensity and vBMD in CTAn software (Preferences → Histogram). All subsequent samples were analyzed under identical imaging and reconstruction parameters, and their grayscale values were converted into calibrated vBMD values using the established calibration curve.

#### Micro-CT scanning for vertebral validation

Samples were scanned using a high-resolution micro-CT system (SkyScan 1276, Bruker, Kontich, Belgium) with a voxel size of 26 μm. The reconstructed images were imported into DataViewer (version 1.7.0.1, Bruker) for alignment and orientation adjustment. In CTAn (version 1.23.0.2+, Bruker), regions of interest (ROIs) were carefully delineated within the trabecular region of the vertebral body for vBMD analysis.

Vertebral samples were analyzed using the same hydroxyapatite phantom–based calibration procedure described above. Briefly, two HA phantoms (0.25 and 0.75 g/cm^3^) were used to establish a linear calibration curve between grayscale intensity and vBMD in CTAn. All vertebral specimens were processed under identical imaging and reconstruction parameters, and grayscale values were converted to calibrated vBMD using the established equation.

#### PCD-CT scanning

Scanning was performed using a PCD-CT system equipped with a cadmium telluride detector (NAEOTOM Alpha, Siemens Healthineers, Germany). Parameters included 120 kV tube voltage, 102 mAs, 0.5 s rotation time, 0.85 pitch, kernel Qr76, and 0.4 mm slice thickness. Raw data were reconstructed into VMIs using syngo.via. ROIs were manually delineated at the same anatomical location as used in the micro-CT analysis to measure the average CT value (HU target) (Figure 6). All samples were prepared using a standardized drilling procedure to create identical and well-defined cylindrical bone defects (8 mm in diameter and 3 mm in depth), and all implanted PEEK scaffolds shared the same dimensions and geometry. These standardized conditions ensured that the defect boundaries were clearly visible in both imaging modalities, allowing the ROIs (diameter 8 mm, thickness 3 mm) to be consistently placed at the same anatomical locations corresponding to the micro-CT analysis. Background ROIs were selected on the same slice, avoiding inhomogeneous regions as much as possible, to record the background signal (HU background), while its standard deviation (SD background) was defined as image noise. ROIs placements were standardized across samples.

Signal-to-noise ratio (SNR) was calculated as:

$$\text{SNR}=\frac{{\text{HU}}_{\text{target}}}{{\text{SD}}_{\text{background}}}$$

Contrast-to-noise ratio (CNR) was calculated as:

$$\text{CNR}=\frac{\left|{\text{HU}}_{\text{target}}-{\text{HU}}_{\text{background}}\right|}{{\text{SD}}_{\text{background}}}$$

#### PCD-CT scanning for vertebral samples

Scanning was performed using a PCD-CT system equipped with a cadmium telluride detector (NAEOTOM Alpha, Siemens Healthineers, Germany). Parameters included 120 kV tube voltage, 102 mAs, 0.5 s rotation time, 0.85 pitch, kernel Qr76, and 0.4 mm slice thickness. Raw data were reconstructed into VMIs using syngo.via. The inferior margin of the vertebral arch was used as the anatomical reference point to ensure spatial correspondence between PCD-CT and micro-CT analyses. Mean CT values of the trabecular region were obtained across 40–80 keV monoenergetic levels for subsequent analysis.

### Quantification and statistical analysis

Statistical analysis was performed using GraphPad (version 10.1.2). Data are presented as mean ± standard deviation. Normality was assessed using the D'Agostino & Pearson test. Two-sided tests were used, and *p* < 0.05 was considered statistically significant.

#### Correlation between CT values and vBMD at different energy levels

Spearman’s rank correlation analysis was performed to assess the monotonic relationship between CT values and micro-CT–derived vBMD across different virtual monoenergetic levels. The resulting correlation coefficients (*r*) were visualized using a heat map.

#### Linear regression analysis at different energy levels

Simple linear regression models were established at each virtual monoenergetic level, with CT values as the independent variable and vBMD as the dependent variable, to characterize their linear relationship. The regression equations and coefficients of determination (*R*^2^) were reported, and scatter plots were generated to visualize the fitting results. Each data point in the scatter plots represents a paired measurement obtained from the same defect site on PCD-CT and micro-CT images.

#### Cross-validation and prediction evaluation at different energy levels

**Figure 7:**
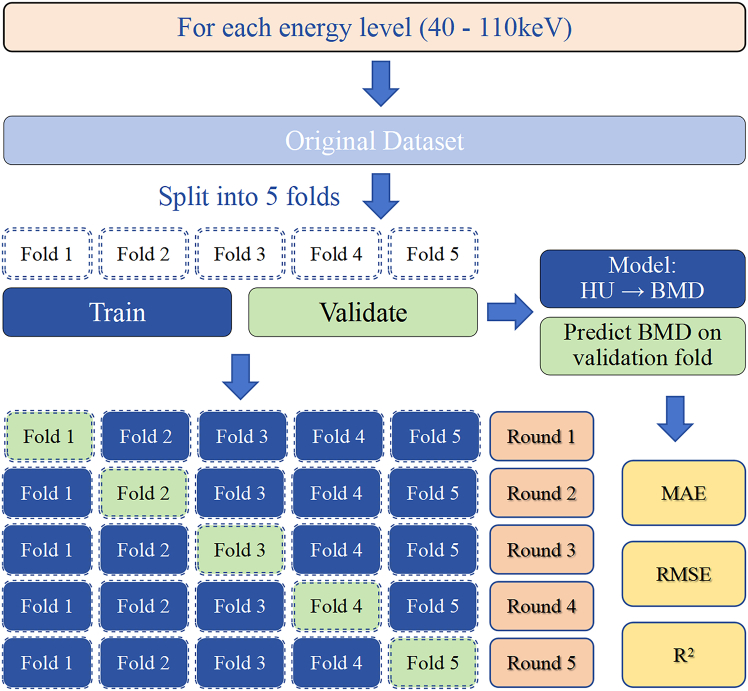
5-fold cross-validation workflow for evaluating the accuracy of vBMD prediction based on PCD-CT-derived VMIs The dataset was randomly split into 5-folds once, and the same grouping was applied across all energy levels. For each round, models were trained on 4-folds and validated on the fifth. Prediction performance was assessed using MAE, RMSE, and *R*^2^.

Five-fold cross-validation was performed to evaluate the stability and accuracy of vBMD prediction across different energy levels (Figure 7). Samples were randomly divided into five subsets using Excel's RAND() function, ensuring consistent grouping across energy levels. In each round, four subsets were used to train a linear regression model, and the remaining subset served as the validation set. This process was repeated five times so that each subset was used once for validation.

Predicted vBMD values were compared with micro-CT-derived vBMD, and the following error metrics were calculated: mean absolute error (MAE), mean square error (MSE), root mean square error (RMSE), and coefficient of determination (*R*^2^). Bland–Altman analysis was also performed to assess agreement, and average bias was calculated.

#### Statistical analysis for vertebral validation

Statistical analysis was performed using GraphPad Prism (version 10.1.2). Data normality was evaluated using the D’Agostino–Pearson test.

##### Correlation analysis

Pearson correlation analysis was conducted to assess the linear relationship between PCD-CT–measured CT values and micro-CT–derived vBMD at different virtual monoenergetic levels. The resulting correlation coefficients (*r*) were visualized as a heat map to illustrate the strength of association across energy levels.

##### Linear regression analysis

Simple linear regression models were established for each monoenergetic level, using CT values as the independent variable and vBMD as the dependent variable, to quantify their linear relationship. Scatter plots were generated to visualize the regression fitting results and correlation strength.

##### Bland-Altman analysis

The CT values obtained from the vertebral samples were substituted into the regression equations previously established from the tibial defect model to calculate the predicted vBMD values. Agreement between the predicted and reference (micro-CT–measured) vBMD values was evaluated using Bland–Altman analysis, in which the mean bias and 95% limits of agreement were computed to assess consistency between the two measurement methods.
