# Supplementary material for: Photon-counting detector CT virtual monoenergetic imaging for bone mineral density quantification: Validation with Micro-CT
Source: iScience. 2026 Mar 3;29(4):115197. doi: 10.1016/j.isci.2026.115197 (PMC13015749; doi:10.1016/j.isci.2026.115197)
Supplement: Document S1. Figures S1–S3 [file mmc1.pdf]

iScience, Volume *29*

Supplemental information

Photon-counting detector CT virtual monoenergetic imaging for bone mineral density quantification: Validation with Micro-CT

Yuanbo Ma, Yaman Li, Danyang Su, Yufang Du, Ke Qi, Simeng Wang, Hao Shen, Mengze Ma, Fei Li, Shenyu Yang, Qiuju Miao, and Xiaopeng Yang

Supplementary Information

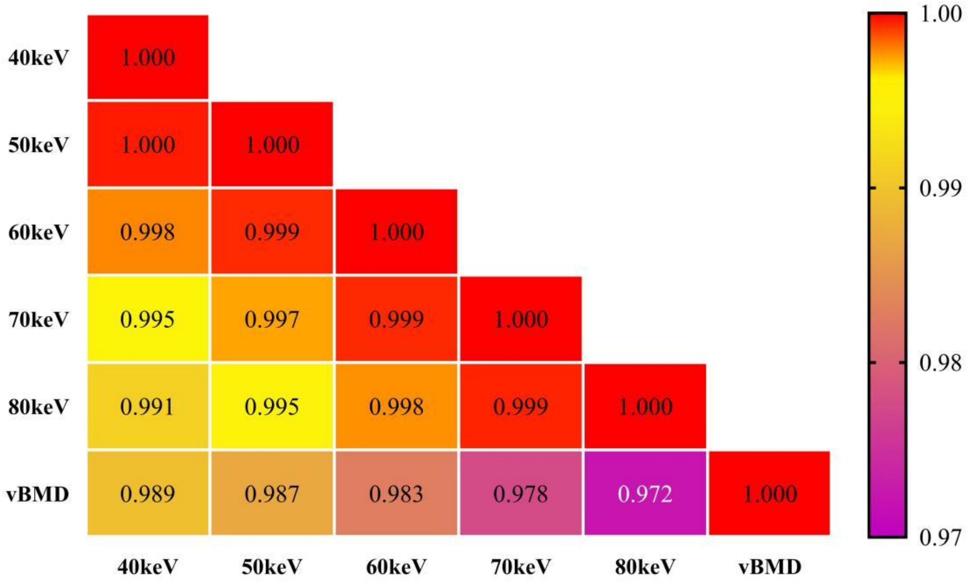

Figure S1. Pearson correlation heat map showing the correlation between CT values and vBMD values in the vertebral trabecular bone region at 40–80 keV, Related to Figure 4. Data are derived from N = 3 biological replicates (rabbits), with 4 vertebral bodies analyzed per animal. The closer the colour is to red, the stronger the positive correlation.

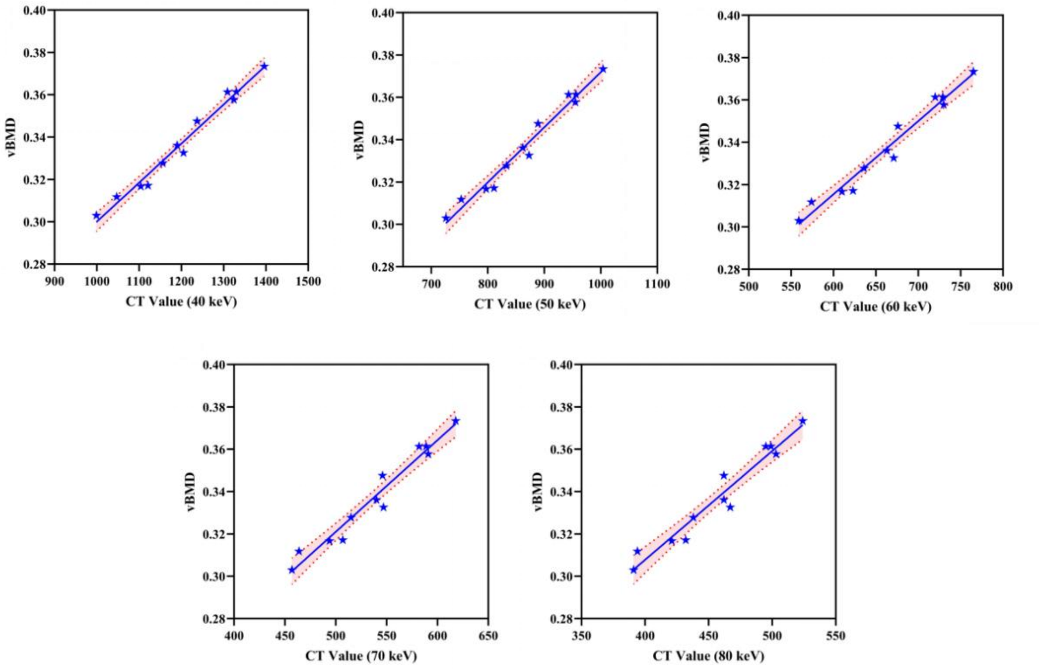

Figure S2. Scatter plots illustrating the linear relationship between CT values obtained

by PCD-CT and vBMD measured by micro-CT in the vertebral trabecular bone region at 40 – 80 keV, Related to Figure 5. Data are derived from N = 3 biological replicates (rabbits), with 4 vertebral bodies analyzed per animal. Each scatter point represents a paired measurement from a single vertebral site.

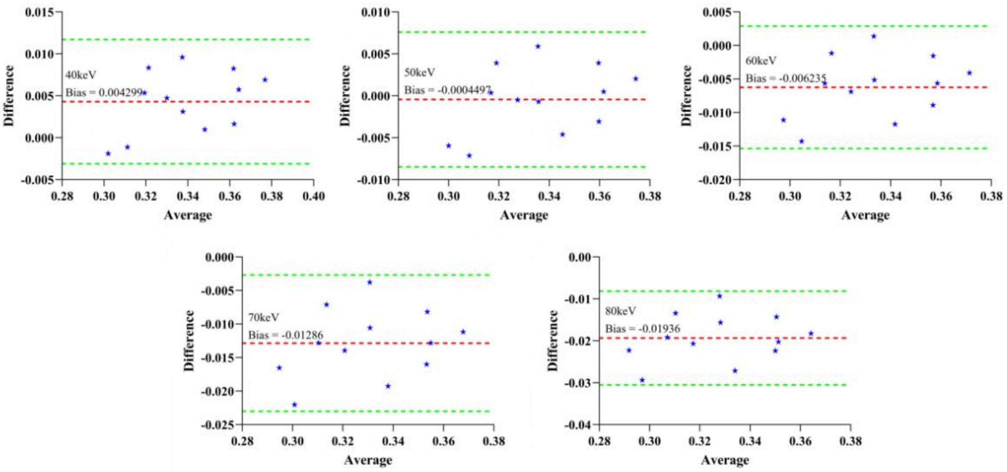

Figure S3. Bland–Altman plots comparing predicted and reference vBMD values of vertebral trabecular bone at 40–80 keV, Related to Figure 7. Data are derived from N = 3 biological replicates (rabbits), with 4 vertebral bodies analyzed per animal. The red dotted line represents the mean bias, and the green dotted lines above and below represent the 95% limits of agreement (±1.96 SD).
